# Unraveling the Compound Eye Design of the Diurnal Moth *Histia flabellicornis* (Lepidoptera: Zygaenidae)

**DOI:** 10.3390/insects16080771

**Published:** 2025-07-27

**Authors:** Qing-Xiao Chen, Ya-Fei Li, Yun-Zhu Huo

**Affiliations:** Henan Province Engineering Technology Research Center of Green Plant Protection, Laboratory of Insect Evolution and Systematics, College of Horticulture and Plant Protection, Henan University of Science and Technology, Luoyang 471000, China; liyafei@stu.haust.edu.cn (Y.-F.L.); huoyunzhu@stu.haust.edu.cn (Y.-Z.H.)

**Keywords:** ommatidium, apposition eyes, visual acuity, ultrastructure, Insecta

## Abstract

Lepidoptera typically exhibit a dichotomy in compound eye design: diurnal butterflies possess apposition eyes for high resolution in bright light, whereas nocturnal moths have superposition eyes for enhanced sensitivity under dim conditions. However, exceptions, particularly among diurnal moths, challenge this pattern and offer insights into the evolution of compound eyes in Lepidoptera. In this study, we investigated the compound eye design of the diurnal moth *Histia flabellicornis* (Fabricius) using light, scanning, and transmission electron microscopy to determine whether it has superposition or apposition eyes, and to quantitatively analyze the structural features and visual acuity. Our results reveal that *H. flabellicornis* possesses apposition-type compound eyes composed of over 2000 ommatidia, each comprising a cornea, a crystalline cone, nine retinula cells forming a fused rhabdom, and a few isolated tracheoles. The calculated interommatidial angles (Δ*ϕ* = 4.08°) and the eye parameter *P* (*P* = 1.74) suggest a visual system adapted to moderate light conditions, balancing spatial resolution with photon capture. These findings confirm the presence of apposition eyes in *H. flabellicornis*, supporting the moth’s adaptation to diurnal behavior and contributing to understanding the evolutionary diversification of compound eye designs in Lepidoptera.

## Simple Summary

The zygaenid moth *Histia flabellicornis* (Fabricius) is a diurnal pest whose larvae feed exclusively on the leaves of *Bischofia polycarpa*, a tree of considerable ecological and cultural importance in East Asian urban landscapes and forest plantations. This study aimed to determine whether the diurnal moth *H. flabellicornis* has apposition or superposition eyes and to evaluate its visual acuity. We found that the moth possesses apposition compound eyes, characterized by direct contact between the crystalline cone and the rhabdom, the absence of a clear zone, nine retinula cells forming one fused rhabdom per ommatidium, and a simplified tracheal system lacking a tapetum. The eyes offer moderate photon capture efficiency under intermediate brightness but provide relatively low spatial resolution. Such eye designs likely support visual function under variable light conditions, such as during dawn and dusk activity or within shaded forest environments. Understanding the visual features of *H. flabellicornis* could contribute to the development of visually based, environmentally friendly pest management strategies for this species.

## 1. Introduction

Lepidoptera, a diverse and widespread group of insects, includes both butterflies and moths, which have adapted to distinct visual ecological niches ranging from bright daylight to moonlight nights [1]. Most butterflies are diurnal and possess apposition compound eyes [2,3,4,5,6], in which individual ommatidia function as isolated optical units to enhance spatial resolution under daylight conditions [7]. In contrast, most moths are nocturnal and have superposition compound eyes that integrate light from multiple ommatidia, thereby maximizing sensitivity in dim environments [8,9,10,11,12]. These two eye designs are traditionally associated with diurnal and nocturnal lifestyles, respectively.

However, several notable exceptions challenge this conventional view. Within butterflies, the nocturnal family Hedylidae possesses superposition eyes, consistent with its nighttime activity [13]. Intriguingly, their sister group, the Hesperiidae, includes some diurnal skipper butterflies that also exhibit superposition eyes [14]. Among moths, certain diurnal species in the noctuid subfamily Agaristinae and the family Sphingidae retain superposition eyes [15,16,17,18]. Conversely, some diurnal moths in the families Sesiidae and Castniidae possess apposition eyes [19,20]. Considering that extant Lepidoptera are hypothesized to have evolved from diurnal ancestors with apposition eyes [7], these exceptions, particularly among diurnal moths, are potentially key to understanding the ancestral visual systems of Lepidoptera.

*Histia flabellicornis* (Fabricius) is a day-flying moth belonging to the family Zygaenidae [21]. Its larvae feed exclusively on the leaves of *Bischofia polycarpa*, a keystone tree species in East Asia known for its vital ecological roles in watershed conservation and microclimate regulation, its economic value as a source of high-quality timber and medicinal resources, and its cultural significance as a traditional symbol in urban landscaping and forest plantations [22]. As a major forestry pest, the larvae of *H. flabellicornis* display conspicuous aposematic coloration and can cause rapid defoliation during outbreaks, leading to severe canopy loss and even plant mortality. Unlike most moths that are nocturnal, adults of *H. flabellicornis* are active during the day for foraging and mating, indicating a distinctive ecological adaptation to diurnal life. However, the visual adaptations that support this behavior remain poorly understood, specifically whether this diurnal moth retains the superposition eyes typical of nocturnal moths or has evolved the apposition eyes characteristic of diurnal Lepidoptera. Understanding the compound eye structure and function in *H. flabellicornis* will not only shed light on visual evolution in moths, but also provide a theoretical basis for the development of species-specific, vision-based pest control strategies.

In this study, we investigated the structural organization of the compound eyes in *H. flabellicornis* using light and electron microscopy to determine the compound eye type. In addition, we performed quantitative analyses to assess the visual acuity of the compound eyes. These anatomical and visual insights are expected to provide valuable information on the visual adaptations of this diurnal moth and the evolutionary diversification of compound eye designs in Lepidoptera.

## 2. Materials and Methods

### 2.1. Insect Collection

Adults of the diurnal moth *Histia flabellicornis* (Fabricius) were collected from the campus of Henan University of Science and Technology, Henan Province, China, in May 2023.

### 2.2. Scanning Electron Microscopy (SEM)

After anesthetizing with diethyl ether, live adults were decapitated using dissecting scissors. The heads were immediately fixed in 2% paraformaldehyde and 2.5% glutaraldehyde in phosphate-buffered saline (PBS, 0.1 M, pH 7.2) at 4 °C for 24 h. The samples were rinsed several times with PBS and cleaned using an ultrasonic cleaner for several seconds. The samples were dehydrated through a graded ethanol series (30%, 50%, 70%, 80%, 90%, 95%, and 100%) for 10 min at each concentration. They were then immersed in ethanol/tertiary butanol mixtures (3:1, 1:1, and 1:3, *v*/*v*) for 15 min each, followed by two changes of 100% tertiary butanol, for 30 min each. After the samples were freeze-dried for 3 h, they were mounted on an aluminum stub using double-sided conductive tape, sputter-coated with gold, and examined using a JSM-IT200 scanning electron microscope (JEOL, Tokyo, Japan) at an accelerating voltage of 15 kV.

### 2.3. Light Microscopy (LM) and Transmission Electron Microscopy (TEM)

Compound eyes were dissected from the heads of anesthetized adults and fixed in 2% paraformaldehyde and 2.5% glutaraldehyde (Structure Probe, Inc., West Chester, NY, USA) in PBS (0.1 M, pH 7.2) at 4 °C until further processing. After several rinses with PBS, the samples were post-fixed in 1% osmium tetroxide (OsO_4_) (TED Pella, Inc., Redding, CA, USA) in the dark for 2 h at room temperature (~25 °C). Following additional rinses, the samples were dehydrated through a graded acetone series (30%, 50%, 70%, 80% for 10 min each; 90% for 15 min; and 100% for 30 min thrice) and infiltrated with the mixtures of acetone/Epon 812 resin (3:1 for 3 h, 1:1 for 5 h, and 1:3 for 14 h), followed by two changes in pure Epon 812 resin (Structure Probe, Inc., West Chester, NY, USA) for 24 h each. The compound eyes were embedded in Epon 812 resin using nadic methyl anhydride as the hardener, dodecenyl succinic anhydride as the softener, and 2,4,6-tri(dimethylaminomethyl)phenol (DMP-30) as the epoxy accelerator. Polymerization was carried out at 30 °C for 24 h and then at 60 °C for 48 h.

For LM, semi-thin sections (2 µm thick) were cut using glass knives on a Leica EM UC7 ultramicrotome (Leica, Nussloch, Germany), stained with 1% toluidine blue solution, and examined under a Nikon Eclipse 80i light microscope (Nikon, Tokyo, Japan).

For TEM, ultra-thin sections (70 nm thick) were cut using a diamond knife on the same ultramicrotome and mounted on 200-mesh formvar-carbon-coated grids. The sections were stained with 2% uranyl acetate for 8 min and 4% lead citrate for 10 min, and examined under a Tecnai G2 Spirit Bio transmission electron microscope (FEI, Hillsboro, TX, USA) at an accelerating voltage of 80 kV.

Morphological terminology for compound eyes followed those presented by Paulus [23] and Kittelmann and McGregor [24].

### 2.4. Data Measurement and Analysis

A total of 40 adult specimens (20 females and 20 males) were used for morphological and anatomical analyses. Sample sizes for specific measurements are indicated as “*n*” in this study. All measurements were performed using ImageJ software (version 1.50i). SEM images were used to quantify compound eye diameters and ommatidial facet diameters (measured as the corner-to-corner distance of hexagonal facets). LM and TEM images were used to measure ommatidial length, corneal thickness, crystalline cone height, and the diameters of the rhabdom and its microvilli. Mean values and standard errors were calculated using SPSS Statistics 20.0 (IBM Corp., Armonk, NY, USA). Sexual differences were evaluated using a two-tailed independent samples *t*-test for datasets with sufficient sample size (*n* ≥ 6) and a non-parametric Mann–Whitney U test when the sample size was small (*n* < 6). Statistical significance was set at *p* < 0.05.

As no significant sexual dimorphism was observed, measurements from both sexes were pooled for optical analyses.

To estimate the visual acuity of the compound eyes, the relevant optical parameters were calculated as follows:

The interommatidial angles (Δ*ϕ*) and the eye parameter *P* (*P*) were determined using the formulas described by Snyder [25] and Land [26]:

Δ*ϕ* = *D*/*R*      *P* = *D*^2^/*R*

where *D* is the facet diameter, and *R* is the local radius of curvature of the eye, calculated from eye segments using the baseline length (*s*) and height (*h*) according to Schwarz et al. [27]:

$$R=\frac{{\left(s/2\right)}^{2}\;+\;h^{2}}{2h}$$

The focal length (*f*) and the lens power (*P_L_*) of the dioptric system were calculated following Schwarz et al. [27]:

*f* = *n*_0_/*P_L_      P_L_ = P_1_ + P_2_ + P_3_*

where the power of the front surface of the lens (*P*_1_), the power of the back surface of the lens (*P*_2_), and *P*_3_ are defined as follows:

*P_1_* = (*n_1_* − *n*_0_)/*r*_1_    *P*_2_ = (*n*_2_ − *n*_1_)/*r_2_    P*_3_ = −*tP*_1_*P*_2_/*n*_1_

Here, *r*_1_ and *r*_2_ are the radii of curvature of the outer and inner corneal surfaces, respectively, and *t* is the corneal thickness. The refractive indices for air (*n*_0_), the corneal lens (*n*_1_), and the crystalline cone (*n*_2_) were set to 1.00, 1.45, and 1.34, respectively.

The *F*-number (*F*) and the rhabdom acceptance angle (Δ*ρ_rh_*) were calculated as follows [25,26]:

*F = f/D*      Δ*ρ_rh_ = d/f*

where *d* is the diameter of the rhabdom.

## 3. Results

### 3.1. External Morphology

**Figure 1:**
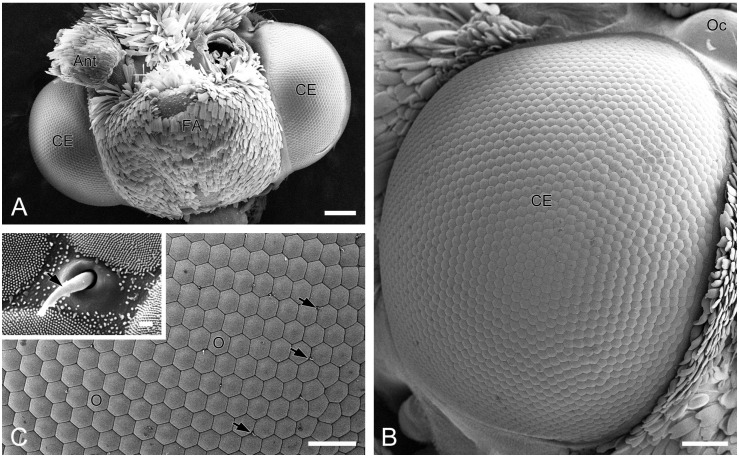
External morphology of compound eyes in *H. flabellicornis*, SEM. (**A**) Frontal view of the head. (**B**) Right compound eye. (**C**) Ommatidia. Arrows indicate sensory hairs. The inset shows a sensory hair (arrow) and numerous corneal nipples on the ommatidial facets. Ant, antenna; CE, compound eye; FA, frontoclypeal area; O, ommatidium; Oc, ocellus. Scale bars: (**A**) = 200 µm; (**B**) = 100 µm; (**C**) = 50 µm; inset of (**C**) = 1 µm.

A pair of compound eyes is located laterally on the head of *H. flabellicornis* (Figure 1A). Each eye is nearly hemispherical, with a diameter of 0.91 ± 0.03 mm in females (*n* = 3) and 0.91 ± 0.02 mm in males (*n* = 3), with no significant sexual dimorphism. Each compound eye comprises over 2000 ommatidia, predominantly hexagonal in shape, with occasional quadrangular and pentagonal ones observed (Figure 1B). The diameter of the ommatidial facet is 24.45 ± 0.30 µm in females (*n* = 8) and 24.43 ± 0.35 µm in males (*n* = 8), with no significant difference. Short sensory hairs occur at the junctions between adjacent ommatidia (Figure 1C). Numerous tiny corneal nipples, which function as anti-reflective nanostructures enhancing light transmission [28], cover the surface of all ommatidial facets (Figure 1C, inset).

### 3.2. Anatomical Structure

**Figure 2:**
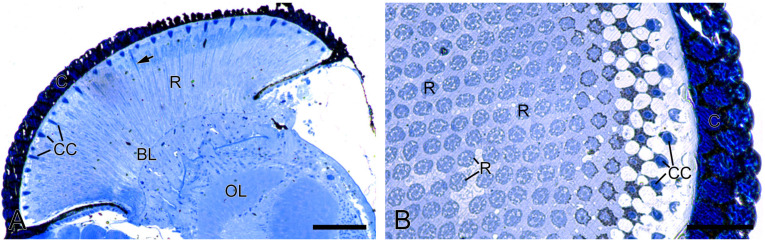
Histological structure of the compound eyes in *H. flabellicornis*, LM. (**A**) Longitudinal section of the compound eye. Arrow points to the rhabdom. (**B**) Transverse section of a compound eye segment. BL, basal lamina; C, cornea; CC, crystalline cone; OL, optic lobe; R, retinula. Scale bars: (**A**) = 100 µm; (**B**) = 50 µm.

The compound eyes of *H. flabellicornis* exhibit a typical apposition-type ommatidial organization, consisting of a cornea, a crystalline cone, a bundle of retinula cells, surrounding pigment cells, and a basal lamina (Figure 2A). In each ommatidium, the crystalline cone lies in direct contact with the underlying retinular complex (Figure 2B).

The total length of the ommatidium was 224.81 ± 5.30 µm in females (*n* = 6) and 238.23 ± 8.16 µm in males (*n* = 6), with no significant difference. Similarly, no significant differences were found in corneal thickness (28.53 ± 0.17 µm in females (*n* = 6), and 29.26 ± 0.40 µm in males (*n* = 6)), crystalline cone height (24.11 ± 0.48 µm in females (*n* = 6), and 22.69 ± 0.73 µm in males (*n* = 6)), or rhabdom diameter (2.64 ± 0.07 µm in females (*n* = 6), and 2.67 ± 0.08 µm in males (*n* = 6)). The above data were used to calculate relevant optical parameters (Table 1).

**Figure 3:**
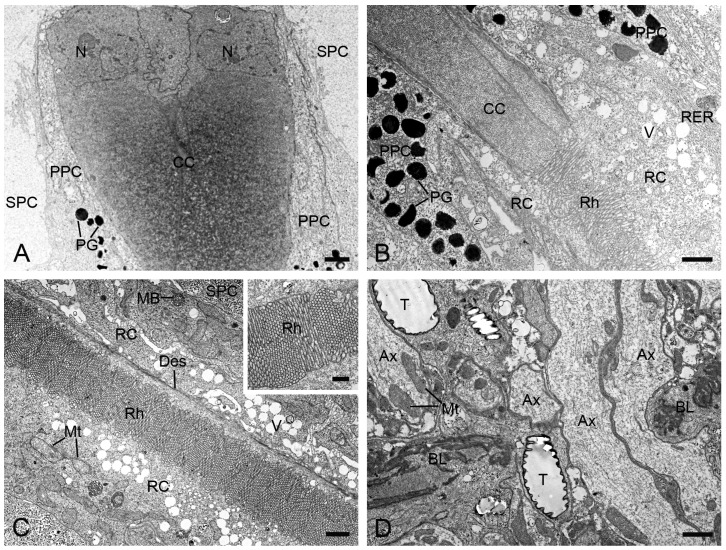
Longitudinal sections of ommatidia in *H. flabellicornis*, TEM. (**A**) Crystalline cone and pigment cells. (**B**) Connecting region of crystalline cone and rhabdom. (**C**) Retinula cells and rhabdom. The inset shows a local rhabdom formed by microvilli. (**D**) Basal lamina. Ax, axon; BL, basal lamina; CC, crystalline cone; Des, desmosome; MB, multivesicular body; Mt, mitochondrion; N, nucleus; PG, pigment granule; PPC, primary pigment cell; RC, retinula cell; RER, rough endoplasmic reticulum; Rh, rhabdom; SPC, secondary pigment cell; T, tracheole; V, vesicle. Scale bars: (**A**–**D**) = 1 µm; inset of (**C**) = 500 nm.

At the distal end of each ommatidium, the cornea consists of approximately 250 thin lamellae. Beneath the cornea, four cone cells, along with their intracellular secretory materials, are arranged radially around the ommatidial axis to form an eucone-type crystalline cone. The nuclei of the cone cells are located in the distal region of the cone (Figure 3A). The crystalline cone tapers proximally and connects directly to the rhabdom (Figure 3B).

**Figure 4:**
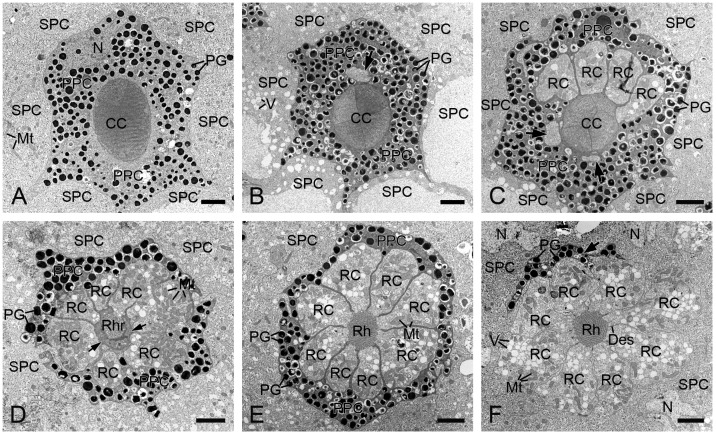
Transverse sections of the distal retinulae in *H. flabellicornis*, TEM. (**A**) Crystalline cone and pigment cells. (**B**) One retinula cell (arrow) appears at the proximal level of the crystalline cone. (**C**) Six retinula cells surround the cone. Arrows indicate two retinula cells at their initial emergence. (**D**) A single rhabdomere from one retinula cell is visible at the cone base (arrows). (**E**) Eight retinula cells contribute their rhabdomeres to a fused rhabdom. (**F**) Nuclei of secondary pigment cells are discernible at the proximal level of the primary pigment cell (arrow). CC, crystalline cone; Des, desmosome; Mt, mitochondrion; N, nucleus; PG, pigment granule; PPC, primary pigment cell; RC, retinula cell; Rh, rhabdom; Rhr, rhabdomere; SPC, secondary pigment cell; V, vesicle. Scale bars: (**A–F**) = 2 µm.

Each ommatidium contains two primary and six secondary pigment cells per ommatidium. The secondary pigment cells extend from the cornea down to the basal lamina, filling the interommatidial spaces (Figure 3A,C,D). Six secondary pigment cells surround an individual ommatidium (Figure 4A–F), with their nuclei located at the proximal level of the primary pigment cells (Figure 4F). The primary pigment cells envelop the crystalline cone throughout the cone length (Figure 3A,B). The primary pigment cells contain abundant electron-dense granules concentrated in the middle to proximal regions (Figure 4A–F). Their nuclei are located at the proximal level of the crystalline cone (Figure 4A).

Beneath the crystalline cone lies the retinula, which consists of nine retinula cells arranged radially (Figure 3B). Each retinula cell specializes its mesial cell membrane into a stack of finger-like microvilli, about 80 nm in diameter (Figure 3C, inset). The microvilli of each retinula cell constitute its rhabdomere. The rhabdomeres of all retinula cells in one ommatidium fuse centrally to form a single rod-shaped rhabdom aligned along the ommatidial axis (Figure 3C). The rhabdoms terminate just above the basal lamina, where the retinula cells turn into axons that contain mitochondria and microtubules (Figure 3D). All axons from each ommatidium converge into a bundle that penetrates the optical lobe through an ostiole (Figure 3D).

**Figure 5:**
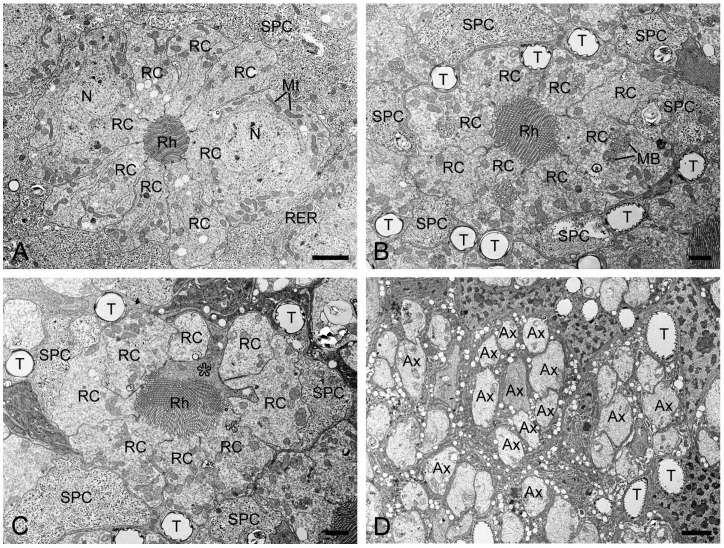
Transverse sections of the middle and proximal retinulae in *H. flabellicornis*, TEM. (**A**) Nuclei of retinula cells are discernible at the middle level of the retinula. (**B**) Two of eight retinula cells cease to contribute to the rhabdom. (**C**) The ninth retinula cell (asterisk) contributes to rhabdom formation. (**D**) Nine axons assemble into a bundle per ommatidium. Ax, axon; MB, multivesicular body; Mt, mitochondrion; N, nucleus; RC, retinula cell; Rh, rhabdom; RER, rough endoplasmic reticulum; SPC, secondary pigment cell; T, tracheole. Scale bars: (**A**) and (**D**) = 2 µm; (**B**) and (**C**) = 1 µm.

In TEM transverse sections, the first retinula cell appears at the proximal level of the crystalline cone, initially without rhabdomeral microvilli (Figure 4B). Further proximally, six retinula cells encircle the cone but still lack rhabdomeres (Figure 4C). At the proximal end of the cone, a single rhabdomere from one retinula cell becomes visible, while the remaining retinula cells do not form the rhabdomeres (Figure 4D). Just below the crystalline cone, eight retinula cells contribute their rhabdomeres to a fused rhabdom (Figure 4E,F). These retinula cells contain abundant mitochondria and abundant electron-lucent vesicles, but few electron-dense pigment granules. Their nuclei are located in the middle region of the retinula, where secondary pigment cells continue to occupy the interommatidial spaces (Figure 5A). At a more proximal level, two of the eight retinula cells do not contribute their rhabdomeres to the rhabdom, so the remaining six retinula cells form the rhabdom (Figure 5B). Approximately four to eight tracheoles are irregularly distributed around each retinula (Figure 5B,C). Eventually, the ninth retinula cell contributes its rhabdomere to the rhabdom (Figure 5C). Near the basal lamina, all retinula cells become axons, with nine axons per ommatidium assembling into a distinct bundle (Figure 5D).

## 4. Discussion

The compound eyes of *H. flabellicornis* are structurally of the apposition type, characterized by direct contact between the crystalline cone and the rhabdom, in contrast to superposition eyes, which feature a clear zone [7]. Apposition eyes are typically associated with diurnal visual ecology of the moth [21].

Visual acuity, determined by interommatidial angles, optical quality, and rhabdom dimensions, is an important criterion for evaluating the spatial resolving capacity of compound eyes [26,29]. In *H. flabellicornis*, the interommatidial angle (*Δϕ* = 4.08°) is larger than that of most flying insects (1–3°) [30], indicating a lower capacity for fine spatial resolution. However, the F-number (*F* = 3.70) falls within the expected range for apposition eyes (typically above 2.1) [31], consistent with a lower light-gathering efficiency under bright conditions [32]. Remarkably, the eye parameter *P* (*P* = 1.74) in *H. flabellicornis* is higher than that of most diurnal insects (0.5–1.0), yet lower than that of nocturnal insects (2–3) [33]. This elevated *p*-value suggests enhanced photon capture, potentially improving contrast sensitivity under moderate to low light [30]. Given that this moth is often active at dawn, dusk, or in dappled forest shade, this combination of relatively limited resolution and enhanced photon capture may reflect a visual compromise to cope with highly variable light environments.

In *H. flabellicornis*, each ommatidium contains nine retinula cells forming a fused rhabdom. This arrangement is consistent with the eye anatomy of the diurnal moth *Paysandisia archon* (Castniidae) [20], and with the apposition eyes of most diurnal butterflies from the families Nymphalidae [5,34,35,36], Lycaenidae [4,6,37], Pieridae [2,38,39], and Papilionidae [3]. The consistent presence of nine retinula cells indicates a conserved anatomical feature in the apposition eyes of diurnal Lepidoptera. In contrast, the ancestral state within Insecta is considered to be eight retinula cells per ommatidium [23,40]. This condition is retained in many extant species such as the predatory bug *Montandoniola moraguesi* (Hemiptera) [41], the scorpionfly *Panorpa dubia* (Mecoptera) [42], the weevils *Eucryptorrhynchus scrobiculatus* and *E. brandti* (Coleoptera) [43], and the sawfly *Arge similis* (Hymenoptera) [44]. According to a recent phylogeny of Lepidoptera [45], early-diverging non-ditrysian lineages such as Micropterigidae, Nepticulidae, and Tischeriidae, as well as basal ditrysian families like Yponomeutidae and Gracillariidae, retain this primitive configuration of eight retinula cells [46,47,48]. Therefore, the presence of nine retinula cells in the highly derived diurnal Lepidoptera may represent a derived condition.

The apposition eyes of *H. flabellicornis* exhibit a simplified tracheal system, with only a few isolated tracheoles extending into the retinal layer. This pattern resembles that observed in *P. archon* [20] and differs markedly from the complex tracheal tapetum typically found in nocturnal moths with superposition eyes [8,9,10,11,12,49]. The absence of a tracheal tapetum suggests that these isolated tracheoles no longer serve a reflective function, but instead serve primarily to supply air to the ommatidia. Unlike the sparse and irregular tracheoles in *H. flabellicornis*, diurnal butterflies possess more consistent and structured tracheal branching patterns, such as the typical “1-4-8” pattern in Nymphalidae and Lycaenidae [4,5,34,35,36], as well as an alternative “1-2-4” pattern in Pieridae [2,50]. Neither of these branching patterns is present in *H. flabellicornis*, whose tracheoles are scattered and irregular, resembling the condition in phylogenetically basal moths such as *Micropterix aruncella* (Micropterigidae) and *Phyllonorycter medicaginella* (Gracillariidae) [48]. This suggests that isolated tracheoles may represent a primitive condition, with organized branching patterns in butterflies being a derived adaptation. However, the simplified tracheal system observed in *H. flabellicornis* is also found in members of the family Papilionidae and certain pierid butterflies [28,50]. This likely reflects a secondary reduction in the more complex tracheal branching system in some diurnal Lepidoptera.

The optical design of most diurnal butterfly apposition eyes is considered afocal, i.e., the proximal region of the crystalline cone functions as an additional lens that refocuses the incoming light into a parallel beam directed toward the rhabdom [51]. The afocal design appears to be also present in *P. archon* [20]. However, anatomical observations alone are insufficient to confirm the presence of such a system. To determine whether the apposition eyes of the diurnal moth *H. flabellicornis* conform to a focal or afocal design, further optical and physiological investigations, such as ray-tracing microtomography [52], intracellular electrophysiological recordings [53], connectomic reconstructions of the visual system [54], and optical coherence tomography [55], are required. In addition, further research that integrates optical analyses with behavioral assays will help elucidate how *H. flabellicornis* uses vision for diurnal activities such as host recognition and mate finding. Such an approach will not only deepen our understanding of visual adaptations in diurnal moths but also provide a scientific foundation for developing more sustainable and ecologically responsible pest management strategies.

## Figures and Tables

**Table 1:** Relevant structural and optical parameters of the compound eyes in *H. flabellicornis*.

Parameters	Data	*n*
Eye radius (*R*)	343.39 ± 16.07 µm	6
Corneal diameter (*D*)	24.44 ± 0.22 µm	16
Outer corneal surface radius (*r*_1_)	13.50 ± 0.47 µm	3
Inner corneal surface radius (*r*_2_)	25.85 ± 1.33 µm	3
Corneal thickness (*t*)	16.78 ± 0.07 µm	3
Rhabdom diameter (*d*)	2.65 ± 0.05 µm	12
Interommatidial angles (Δ*ϕ*)	4.08°	−
Eye parameter *P* (*P*)	1.74	−
Focal length (*f*)	90.48 µm	−
*F*-number (*F*)	3.70	−
Rhabdom acceptance angle (Δ*ρ_rh_*)	1.68°	−

Data are presented as the mean ± SE. *n* indicates the sample size. “−” indicates a calculated value without an associated sample size.

## Data Availability

The data presented in this study are available on request from the corresponding author due to privacy and legal reasons.
